# Polydopamine/Montmorillonite-Based Brushite Calcium Phosphate Cements: Synergize Mechanical and Osteogenic Capacity for Bone Regeneration

**DOI:** 10.34133/bmr.0213

**Published:** 2025-06-09

**Authors:** Yukang Gong, Yanan Niu, Chenhao Wang, Gaoqi Ye, Menghang Chen, Gaokai Hu, Yang Hu, Yuhan Xue, Tao Guo, Wenjuan Yin, Yuangong Zhang, Zheng Gao, Feng Liu, Wenshan Gao

**Affiliations:** ^1^ School of Clinical Medicine, Hebei University, Baoding 071002, Hebei, China.; ^2^ Affiliated Hospital of Hebei University, Hebei University, Baoding 071002, Hebei, China.; ^3^ School of Basic Medical Sciences, Hebei University, Baoding 071002, Hebei, China.

## Abstract

Although brushite calcium phosphate cements (Bru-CPCs) possess good bioactivity and biocompatibility, their low compressive strength hinders their effective application in osteoporotic bone defect treatment. Therefore, the aim of this study was to improve the compressive strength without sacrificing the osteogenesis properties of Bru-CPCs. Montmorillonite (MMT) was added into Bru-CPCs to get Bru-CPCs/1.5% MMT. The compressive strength of Bru-CPCs/1.5% MMT increased to 13.31 MPa, but the interfacial interactions between MMT and Bru-CPCs (inorganic–inorganic phase) limited further improvement in compressive strength. Inspired by the adhesive proteins in mussels, MMT was coated with a polydopamine (PDA) layer to get MMT@PDA. The compressive strength of Bru-CPCs/1.5% MMT@PDA further increased to 16.58 MPa. Then, the surface morphology, adhesiveness, biocompatibility, and bioactivity of CPCs/1.5% MMT@PDA were evaluated. All data indicated that by adding MMT@PDA, the compressive strength was improved without sacrificing the osteogenesis properties of Bru-CPCs.

## Introduction

Large bone defects caused by bone trauma, infection, surgery, or other systemic diseases cannot achieve healing without bone graft [1–3]. Autologous bone grafts and allografts can no longer meet the current clinical needs. Therefore, biomaterials are in urgent need as bone grafts [4,5]. Calcium phosphate cements (CPCs), with similar constituents as bone tissue, possessing good bioactivity and biocompatibility, are widely used clinically as bone fillers [6–8]. As a member of CPCs, brushite calcium phosphate cements (Bru-CPCs) are widely studied for their injectability, molding, and degradable properties [9–11]. However, their compressive strength and osteogenesis properties need to be improved before use in the long-term remodeling of bone [11–13].

One efficient strategy to address the above problem is to add metal ions, nanoclay, polymers, or protein growth factors into Bru-CPCs [14–16]. Silicate-related materials like nanoclay and ceramic have attracted much attention and shown great potential for bone tissue engineering [17–19]. Montmorillonite (MMT) is a kind of silicate nanoclay, which has been approved by the Food and Drug Administration for application to medicinal products. Extensive studies have shown the potential of MMT as tissue engineering scaffolds [20,21]. The addition of MMT into biomaterials could improve their thermal and mechanical properties, as well as enhance their osteogenic bioactivity [22,23]. Cui et al. [22] prepared methacrylated glycol chitosan–MMT nanocomposite hydrogels for bone tissue engineering. The Young’s modulus of the nanocomposite hydrogels increased from 10 to 60 kPa with the addition of 4% MMT. Sun et al. [24] prepared strontium-doped MMT coating on Mg–Ca alloy as bone implants. In vitro studies on cell activity, alkaline phosphatase (ALP) activity, and cell morphology confirmed that the Sr–MMT coating had satisfactory biocompatibility and improved the osteogenic bioactivity.

Besides the attractive advantages, such as low cost, wide source, and stable properties, MMT contains a wealth of trace elements (Ca, Si, Mg, Li, and Al) essential for osteogenesis, which can be expected to enhance bone formation by releasing these active ions. Tong et al. [23] developed an MMT-reinforced bone scaffold and demonstrated that adding MMT to hydrogels markedly improved scaffold mechanics. Osteogenic differentiation and mineralization deposition were also improved by incorporating MMT. Thus, the introduction of MMT seemed a potential strategy to improve the mechanical properties and osteogenic bioactivity of Bru-CPCs.

MMT has a 2-dimensional layered structure. Polymer chains can easily bind with MMT or intercalate into the layer to form a 3-dimensional network structure, to give strong interfacial interaction between MMT and the polymer scaffold (organic–inorganic phase), which is beneficial for mechanical properties [25]. When introducing MMT into Bru-CPCs, the weak interfacial interaction between the inorganic–inorganic phase may hinder the mechanical properties and osteogenic bioactivity of Bru-CPCs. Therefore, surface engineering of MMT could be an effective road map to solve the above problem. Mussel-inspired polydopamine (PDA) can afford extraordinary adhesion to modify the poor interfacial interactions [26,27].

Herein, to modify the poor interfacial interactions between MMT and Bru-CPCs and improve the mechanical properties and osteogenic bioactivity of MMT-based Bru-CPCs, MMT was coated with a PDA layer to get MMT@PDA. Then, MMT@PDA was added into Bru-CPCs to prepare composite bone cement, Bru-CPC/MMT@PDA. Owing to the adhesion of PDA, the interfacial interactions of MMT and Bru-CPCs were improved, thereby enhancing the mechanical properties of Bru-CPC/MMT@PDA. The physicochemical properties, surface morphology, adhesiveness, biocompatibility, and bioactivity were evaluated. All data indicated that the prepared MMT@PDA here described can be an excellent candidate for bone tissue engineering.

## Materials and Methods

### Materials

MMT (pharmaceutical grade) and 2-(3,4-dihydroxyphenyl)ethylamine hydrochloride were purchased from Energy Chemicals. β-Tricalcium phosphate (β-TCP, Ca_3_(PO_4_)_2_, 600 to 900 mesh, 97.5%, medical grade, 3A) and monocalcium phosphate monohydrate (MCPM, Ca(H_2_PO_4_)_2_·H_2_O, 98%, 3A) were purchased from Energy Chemicals.

### Preparation of MMT@PDA

Five grams of MMT was taken and added to a round-bottom flask; then, 200 ml of distilled water was added, a magnetic stir bar was put in, and the mixture was ultrasonically stirred for 30 min. The Tris solution and 2-(3,4-dihydroxyphenyl)ethylamine hydrochloride were taken out from the refrigerator and allowed to reach room temperature. Then, 2 g of dopamine was added. The pH value was adjusted to an alkaline level, and the mixture was stirred for 48 h. It was centrifuged and washed with water, and a precipitate was obtained at 8,000 r/min. It was freeze-dried to obtain dry MMT@PDA.

### Thermal gravimetric analysis of MMT@PDA

Thermal gravimetric analysis (TGA) measurement was carried out under nitrogen on PerkinElmer Pyris 6 TGA (heating rate of 10 °C/min) to record TGA curves.

### Preparation of CPCs

CPCs are composed of 2 calcium phosphate powders as the solid phase and the liquid phase. The solid phase is composed of β-TCP and MCPM (ratio 6/4) and MMT or MMT@PDA. The liquid phase is a 0.5 M citric acid solution. The liquid–solid ratio is 0.7 ml/g. The samples are represented as Bru-CPCs, Bru-CPCs/1.5% MMT, and Bru-CPCs/1.5% MMT@PDA, respectively. The slurry prepared under the above conditions was filled into a cylindrical polytetrafluoroethylene mold with Φ 4.6 × 6 mm. After 5 min, the mold was demolded, and the sample was placed in a constant-temperature and constant-humidity chamber (37 °C, 100% humidity) for 48 h.

### Setting time

The setting time of CPCs was determined by a Gilmore apparatus compliant with ASTM C266-13. The Gilmore apparatus included an initial setting indenter (113.4 ± 0.5 g, Φ2.12 ± 0.05 mm) and a final setting indenter (453.6 ± 0.5 g, Φ1.06 ± 0.05 mm). After the solid powder was mixed with the setting liquid, the paste was transferred into a mold (diameter of 7.5 mm and height of 15.0 mm) and placed at 37 °C and 98% relative humidity. The indenters were lowered vertically on the cement surface for 5 s to determine the setting time at intervals of 30 s until the paste was hardened. The initial and final settings occurred when there was no complete cyclic penetration using the corresponding indenters. Each test was repeated 5 times.

### Injectability property

The injectability of CPC was tested as follow: 1 g of the as-prepared paste was put into a 5-ml syringe with an opening needle with a diameter of 1.5 mm. After 8 min, a force of 50 N was applied vertically at the syringe piston with a crosshead speed of 2 mm/min using a universal tester (Shandong Wanchen Testing Machine Co., LTD, CMT2503). The injectability of CPC was calculated using Eq. 1:

$$\text{Injectability}=\left(m1-m2\right)/\left(m1-m0\right)$$ (1)

where *m*0 is the weight of the syringe, *m*1 is the initial weight before injection, and *m*2 is the weight after injection. For the injectability test, 5 samples were used as duplicates, and the results are expressed as a mean ± standard deviation (SD).

### Compressive strength of CPCs

The compressive strengths of CPCs were tested in a universal tester (Shandong Wanchen Testing Machine Co., LTD, CMT2503). The crosshead speed was 0.5 mm/min. Five samples were tested for each group. The compressive strength was finally expressed as mean ± SD.

### Scanning electron microscopy and X-ray diffraction

The fractured surfaces of CPCs were observed by scanning electron microscopy (SEM; Phenom ProX, Phenom China). All samples were precoated with Au-Pd coating and then observed by SEM. SEM analysis was performed on the cross-sections of the fractured samples. X-ray diffraction (XRD) analysis was performed using Cu Kα radiation under the following conditions: an operating voltage of 40 kV, a current of 40 mA, a scanning range of 2*θ* = 5° to 60°, and a scanning speed of 6°/min.

### Cell culture

Mouse embryo osteoblast precursor cells (MC3T3-E1, Procell Life Science&Technology Co., Ltd., Wuhan, China) were cultured in alpha minimum essential medium (α-MEM; Biological Industries, Israel) with 10% fetal bovine serum (FBS; Biological Industries, Israel) and 1% penicillin–streptomycin solution (Solarbio, China). Cell digestion was performed by using trypsin EDTA solution (Solarbio, China). The cells were cultured in a normal cell incubator environment (37 °C, 5% CO_2_, and 95% air). For osteogenic induction studies, the MC3T3-E1 cells were cultured in α-MEM for 2 d. When the proliferation reached 70% to 80%, the cell medium was replaced with osteogenic inducing medium (OIM). OIM was prepared by adding 10% FBS, 1% penicillin–streptomycin solution, 10 mM β-glycerophosphate (Sigma-Aldrich), 50 μM ascorbic acid (Sigma-Aldrich), and 100 nM dexamethasone (Sigma-Aldrich) into α-MEM. All of the cement specimens used in this study were sterilized by soaking in 75% alcohol.

### Cell adhesion and cell viability

The MC3T3-E1 cells were cultured and seeded on tablet samples (4.6 mm × 6 mm) of each cement group with 5 × 10^4^ cells per well in 24-well tissue culture plates. The cells were cultured at 37 °C in a humidified environment. After 24 h, the basal medium (BM) was removed and the cells were washed 3 times with phosphate-buffered saline (PBS) and then treated with 4% paraformaldehyde for 15 min at room temperature. The cells were dehydrated in different grades of ethanol (30%, 50%, 70%, 90%, 95%, and 100% ethanol in water, dehydrated for 5 min twice with each grade) and dried in a freeze dryer. The cell samples were coated with gold and tested by SEM (Phenom ProX, Phenom China). The cell adhesion was observed by SEM.

The cell viability was evaluated by cell proliferation assay and live/dead staining assay. The proliferation was assessed by Cell Counting Kit-8 (CCK-8) assay (Solarbio, China). Briefly, Bru-CPCs, Bru-CPCs/1.5% MMT, and Bru-CPCs/1.5% MMT@PDA samples (4.6 mm in diameter× 6 mm in height) were immersed in BM with 10% FBS and put in a cell incubator for 24 h in a normal cell incubator environment (37 °C, 5% CO_2_, and 95% air). Then, cement extracts with an extraction ratio (surface area of samples/volume) of 1.25 cm^2^/ml were obtained, according to ISO 10993. Each well was seeded with 5 × 10^3^ cells and incubated in α-MEM with 10% FBS in 96-well plates. After 24 h, the cell medium was displaced with the cement extracts. With additional culture times of 1, 2, and 3 d, respectively, the extracts in plates were replaced with fresh 90 μl of BM and 10 μl of CCK-8 for each well. After incubation for another 3 h in the cell incubator, the absorbance was measured at 450 nm by Multiskan Spectrum Microplate Spectrophotometer (Thermo Scientific). The cell medium without extract was used as the control.

The MC3T3-E1 cells were cultured and seeded as done in the above process by using 5 × 10^4^ cells per well in 96-well tissue culture plates. After incubation for 24 h, the cell medium was displaced with the cement extracts and incubated for another 24 h. The Live/Dead Viability/Cytotoxicity Kit (Beyotime, C2015M) was used according to the manufacturer’s instructions. Briefly, after the cell culture medium was removed, PBS was used to wash twice and calcein AM/propidium iodide was added to stain live and dead cells, respectively. After incubating for 30 min in a normal cell incubator environment, the BM was removed and the cells were washed 3 times with PBS; the cells were then examined via a confocal microscope (FV3000 LCM, Olympus).

### Osteogenic differentiation

The MC3T3-E1 cells were cultured with sterilized cement samples (4.6 × 6 mm) in 6-well plates with 1 × 10^5^ cells per well in BM. The cell cultures were maintained in a normal cell incubator environment (37 °C, 5% CO_2_ and, 95% air). The BM was changed daily, and the side was washed 2 times with PBS. After the cell confluence reached approximately 70% to 80%, the culture medium was changed into OIM to induce differentiation of MC3T3-E1. After being incubated in OIM for 7 d, OIM was removed and the cells were washed 2 times with PBS. Differentiation of MC3T3-E1 was tested with an ALP activity test.

ALP activity was examined with an ALP detection kit (Solarbio, BC2145). After 7 d of incubation, 0.1% Triton X-100 was added and 30 μl of cell lysate was used for ALP activity detection, and the ALP assay referred to the kit protocol (Beyotime Biotechnology). The total cellular protein was quantified simultaneously by a bicinchoninic acid protein assay kit (Thermo Fisher Scientific). The ALP activity results were normalized by total cellular protein. The culture medium without cement sample was used as the control.

After 7 d of incubation, the ALP expression of MC3T3-E1 at day 7 was detected by staining with BCIP/NBT Alkaline Phosphatase Color Development Kit (Beyotime, China) following the manufacturer’s instructions. Generally, MC3T3-E1 cells on the disks were fixed by 4 vol% poly-formaldehyde solution for 30 min and stained by BCIP/NBT solution (250 μl) for 15 min. The culture medium without cement sample was used as the control. Images of staining samples were captured by an optical microscope.

### Expression of the osteogenesis-related genes

The osteogenesis-related genes of MC3T3-E1 treated with cement in OIM were tested by quantitative reverse transcription polymerase chain reaction (qRT-PCR). The MC3T3-E1 cells were cultured with sterilized cement samples in 6-well plates with 1 × 10^5^ cells per well in BM. The BM was changed daily, and the side was washed 2 times with PBS. After the cell confluence reached approximately 70% to 80%, the culture medium was changed into OIM to induce differentiation of MC3T3-E1. After being treated for 7 and 14 d, the cells were digested by EDTA (Solarbio, China); RNA was extracted from the cell samples with Easyspin RNA Rapid Tissue and Cell Kit (RA105-1, BMBiomed, China) after cell sample collection.

The RNA reverse-transcribed into complementary DNA was used by PrimeScript RT Agent Kit with gDNA Eraser (Takara, Japan). qRT-PCR was performed to examine the gene expression levels of Runx2, SPP1, Col-I, IBSP, and Bglap. These genes were quantified in real time by TB Green series (Takara, Japan). The primer sequences’ information is listed in Table. All quantifications were normalized to the endogenous control glyceraldehyde 3-phosphate dehydrogenase. These procedures were performed in a PCR machine (Thermo ABI7300 real-time PCR machine, New York, USA). Relative gene expression levels were determined using the 2^−ΔΔCt^ method.

**Table.** The sequences of genes

Gene	Primer sequences (5′ to 3′)-F	Primer sequences (5′ to 3′)-R
Gapdh	CGGGGTCCCAGCTTAGGTTC	ATCCGTTCACACCGACCTTC
Runx2	TCACCTTGACCATAACAGTCTTCA	GGCGGGACACCTACTCTCAT
SPP1	AGCAAGAAACTCTTCCAAGCAA	GTGAGATTCGTCAGATTCATCCG
Col-I	GCTCCTCTTAGGGGCCACT	CCACGTCTCACCATTGGGG
IBSP	CAGGGAGGCAGTGACTCTTC	AGTGTGGAAAGTGTGGCGTT
BgIap	GGCAATAAGGTAGTGAACAG	CAAGCCATACTGGTCTGATA

F, forward primer; R, reverse primer

### Statistical analysis

Quantitative data were provided as mean ± SD. Statistical analysis was performed using a one-way analysis of variance, followed by Tukey’s posttest comparison (GraphPad Software 8.0, USA). In all cases, the results were considered statistically significant with a *P* value <0.05 (*P < 0.05, **P < 0.01, and ***P < 0.001).

## Results

### Thermal performance analysis of MMT@PDA, setting time, injectability, compressive performance fracture morphology characterization, and XRD of bone repair materials

**Fig. 1.**
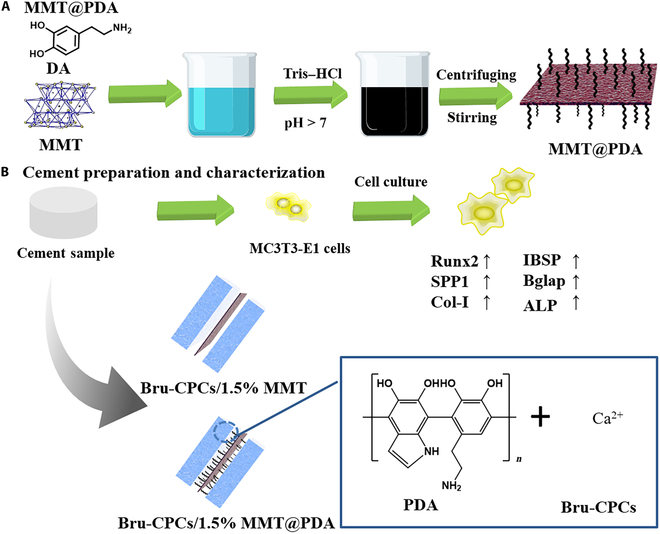
Schematic diagram of MMT@PDA (A) and cement preparation and characterization (B). MMT, montmorillonite; PDA, polydopamine; DA, dopamine; Bru-CPCs, brushite calcium phosphate cements.

**Fig. 2.**
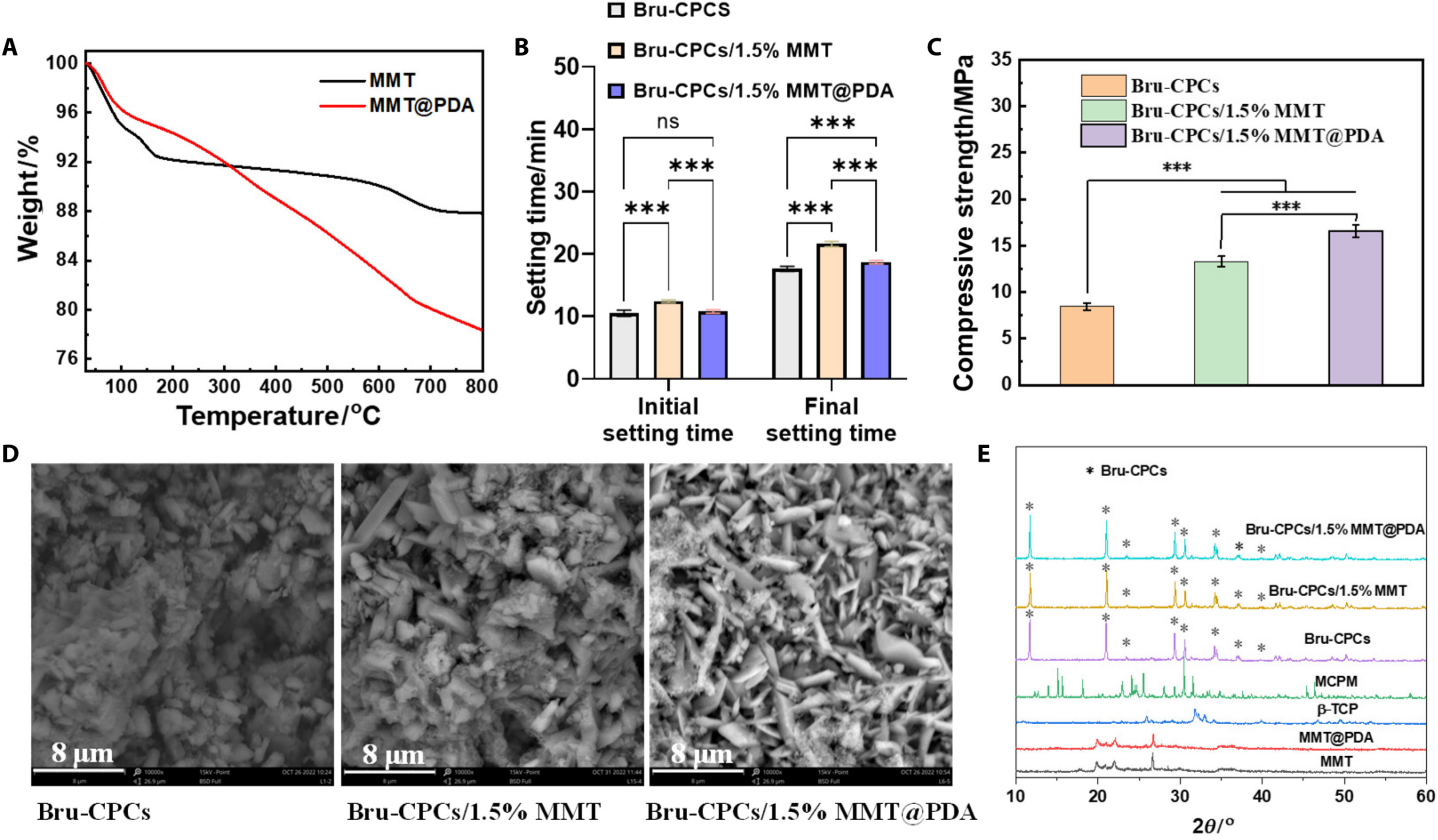
(A) Thermal gravimetric analysis (TGA) curves of MMT and MMT@PDA. (B) Setting time of CPCs (***P < 0.001). (C) Compressive strength of bone repair materials (incubated at 37 °C and 100% relative humidity for 2 d, ***P < 0.001). (D) Morphology of bone repair materials after mineralization. (E) X-ray diffraction (XRD) of MMT, MMT@PDA, β-tricalcium phosphate (β-TCP), monocalcium phosphate monohydrate (MCPM), Bru-CPCs, Bru-CPCs/1.5% MMT, and Bru-CPCs/1.5% MMT@PDA.

MMT@PDA, as a black powder, was synthesized in an alkaline environment as shown in Fig. 1A. PDA can be grafted on MMT owing to its redox activity and binding abilities. The grafting rate of PDA on MMT was studied by thermogravimetric analysis (Fig. 2A). As seen from Fig. 2A, the weight decreased upon increasing the temperature from ambient temperature to 800 °C, with weight loss occurring in 2 stages. The first stage begins at 55 °C, corresponding to the loss of water. The second stage starts at approximately 170 °C and ends at 660 °C, corresponding to PDA decomposition. After 800 °C, the remaining weight was 87.7% and 78.3% for MMT and MMT@PDA, respectively, which indicates that PDA has been successfully grafted onto MMT. After PDA functionalization, MMT and MMT@PDA were used as solid additives to be incorporated into Bru-CPCs to obtain Bru-CPCs/1.5% MMT and Bru-CPCs/1.5% MMT@PDA, respectively. The preparation process is shown in Fig. 1B.

Bru-CPCs exhibited an initial setting time of 10.5 min and a final setting time of 18 min (Fig. 2B). For Bru-CPCs/1.5% MMT, the initial setting time was 12.5 min, and the final setting time was 21.5 min. In the case of Bru-CPCs/1.5% MMT@PDA, the initial setting time was 11 min, and the final setting time was 19 min. The injectabilities of Bru-CPCs, Bru-CPCs/1.5% MMT, and Bru-CPCs/1.5% MMT@PDA were about 95%, 93%, and 94%, respectively. Figure 2C shows the compressive strength of pure Bru-CPCs, Bru-CPCs/1.5% MMT, and Bru-CPCs/1.5% MMT@PDA bone cements which were molded to obtain cylindrical forms (Fig. 1B). The compressive strength of pure Bru-CPCs, measured after curing in a constant-temperature and constant-humidity chamber (37 °C, 100% humidity) for 48 h, was 8.46 MPa, and the result was consistent with the performance reported in the literature [12]. The compressive strength of Bru-CPCs/1.5% MMT increased to 13.31 MPa with the addition of 1.5 wt% MMT. The result showed that the addition of MMT can improve the weak compressive strength of bone repair materials. After adding 1.5 wt% MMT@PDA, the compressive strength of Bru-CPCs/1.5% MMT@PDA further increased to 16.58 MPa, indicating that PDA can modify the interfacial interactions with further improvement in the compressive strength of bone repair material.

The fracture morphology of bone cement was characterized by SEM analysis (as shown in Fig. 2D). The fracture morphology of pure Bru-CPCs was irregular platelike or flaky brushite crystals with micron to nanometer sizes. Compared with that of pure Bru-CPCs, due to the addition of MMT, which provides crystal nuclei, the platelike crystallization of Bru-CPCs/1.5% MMT was more regular. After introducing MMT@PDA, the crystals of Bru-CPCs/1.5% MMT@PDA are more closely and regularly stacked, and the grain size becomes smaller, which is beneficial to improving the mechanical properties. The XRD patterns of the as-prepared materials are shown in Fig. 2E. The dominant crystalline phase in the final product was identified as Bru-CPCs (JCPDS no. 72-0713). The results indicated that the addition of MMT or MMT@PDA did not alter the chemical reaction between β-TCP and MCPM, as the characteristic peaks of Bru-CPCs remain consistent and unaffected by the incorporation of these additives. This suggested that the addition of MMT or MMT@PDA did not interfere with the formation of the desired crystalline phase.

### In vitro cell adhesion and proliferation of MC3T3-E1 cells on the bone repair materials

**Fig. 3.**
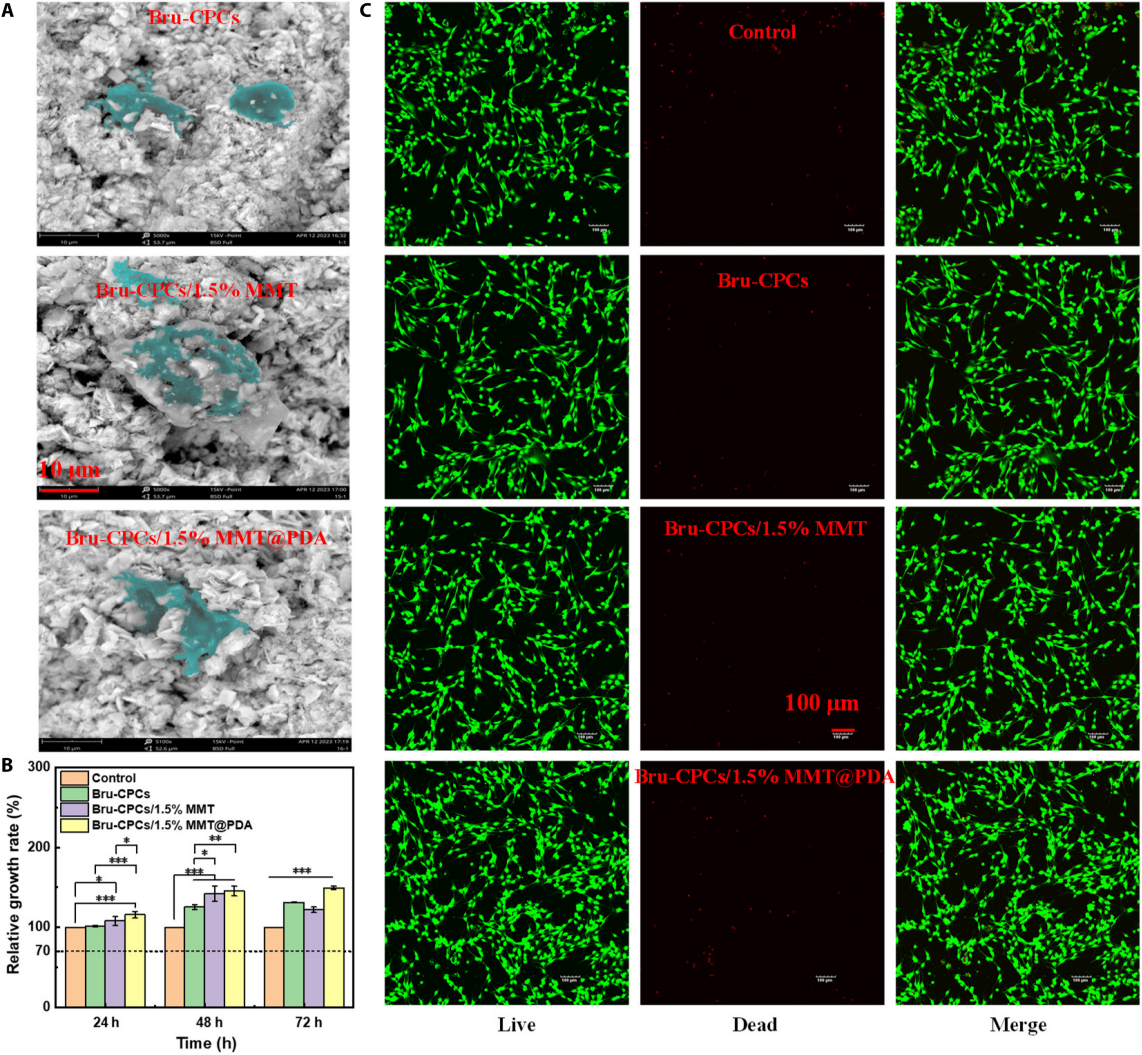
(A) Scanning electron microscopy (SEM) images of bone repair materials cultured with cells for 24 h. (B) Cell viability of bone repair materials, Cell Counting Kit-8 (CCK-8) test (*P < 0.05, **P < 0.01, and ***P < 0.001). (C) Live/dead staining images of bone repair materials after culturing with cells for 24 h.

Cell adhesion was the first and most basic process of the interaction between cells and bone repair materials. The adhesion and spreading of MC3T3-E1 cells on Bru-CPCs, Bru-CPCs/1.5% MMT, and Bru-CPCs/1.5% MMT@PDA after MC3T3-E1 cells and bone repair materials were cultured for 24 h are shown in Fig. 3A. As can be seen from the SEM images, the morphology of MC3T3-E1 cells on bone repair materials was clear, showing an extended state of sheetlike and filamentous feet. This indicates that the addition of MMT and MMT@PDA does not affect the adhesion growth and maintenance of morphology of MC3T3-E1 cells on bone repair materials.

Cell viability was studied by CCK-8 analysis (Fig. 3B) and live/dead staining (Fig. 3C). CCK-8 tests were performed on 1, 2, and 3 d, respectively. As can be seen from Fig. 3B, the relative survival rates of cells were all higher than 70% (the cell survival rate of the control group is defined as 100%). After culturing for 1 d, the survival rates of cells cultured with the extract of Bru-CPCs, Bru-CPCs/1.5% MMT, and Bru-CPCs/1.5% MMT@PDA samples were 101%, 108%, and 115%, respectively. After culturing for 3 d, the survival rates of cells cultured with the extract of Bru-CPCs, Bru-CPCs/1.5% MMT, and Bru-CPCs/1.5% MMT@PDA samples were 131%, 122%, and 149%, respectively. This shows that the addition of MMT does not affect the biocompatibility of bone repair materials. The addition of MMT@PDA increases the cell proliferation rate to a certain extent and improves the biocompatibility of bone repair materials.

The results of live/dead staining analysis further verified the results of CCK-8 analysis. As can be seen from Fig. 3C, there was only a little red fluorescence in the 4 components. Compared with the control group and the pure Bru-CPCs group, with the addition of MMT and MMT@PDA, the cell density in the Bru-CPCs/1.5% MMT group and the Bru-CPCs/1.5% MMT@PDA group increased, indicating that the addition of MMT and MMT@PDA effectively improved the biocompatibility of bone repair materials and could promote cell proliferation to a certain extent.

### In vitro osteogenic effect of MC3T3-E1 cells on the bone repair materials

**Fig. 4.**
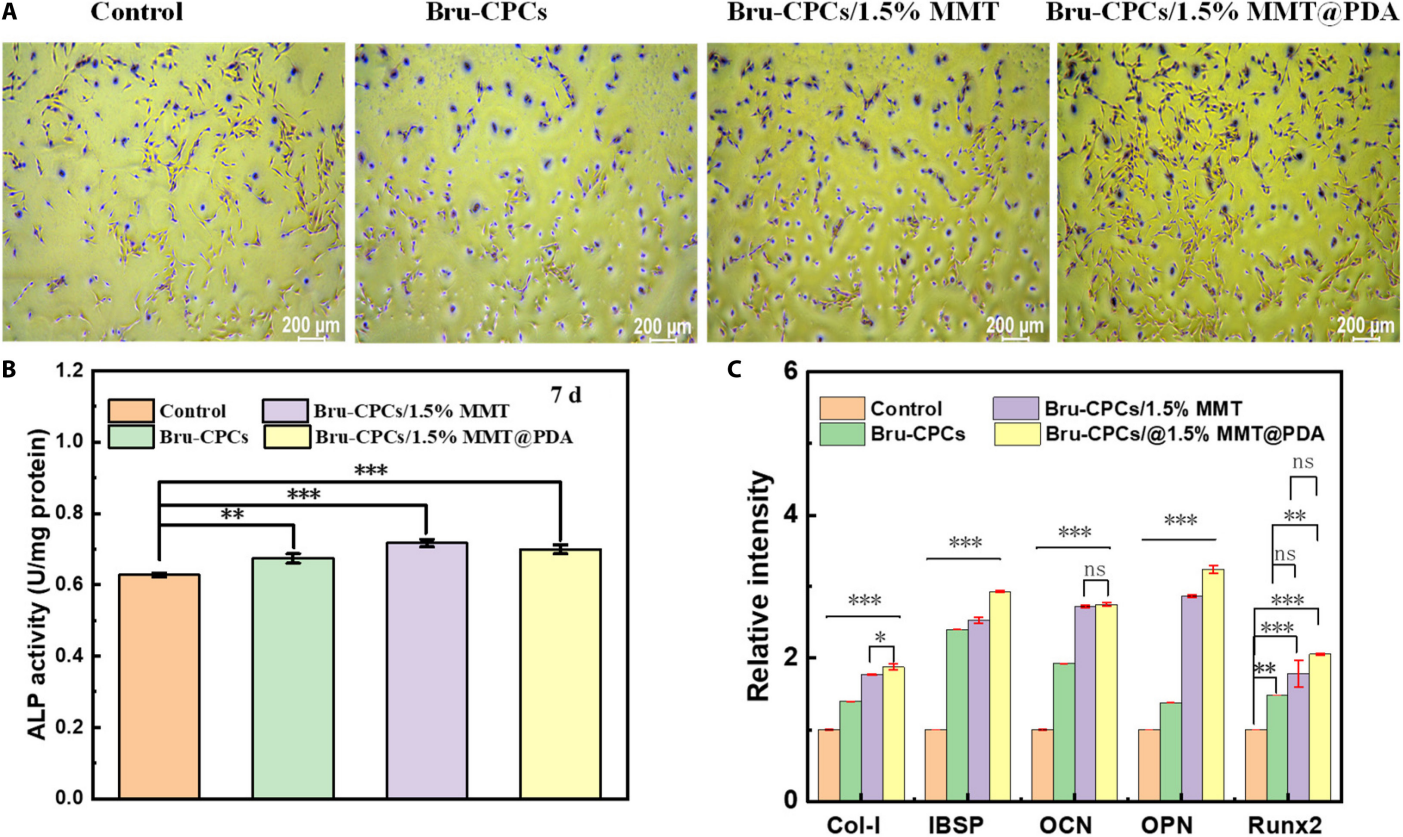
(A) Alkaline phosphatase (ALP) staining of bone repair materials. (B) ALP activity of bone repair materials (*P < 0.05, **P < 0.01, and ***P < 0.001). (C) Osteogenesis-related gene expression test of bone repair materials (*P < 0.05, **P < 0.01, and ***P < 0.001).

ALP, as an early osteogenic marker, was mainly distributed in membrane-bound transport proteins and promotes cell maturation and calcification. Quantitative detection of ALP can reflect the differentiation level of osteoblasts. The higher its activity, the more obvious the differentiation of preosteoblasts into mature osteoblasts. The osteogenic induction of bone cement was determined by ALP staining of samples after 7 d of cultivation. The ALP expressions of all samples and the control group MC3T3-E1 were positive (Fig. 4A), indicating that all bone repair materials have the potential to promote osteogenesis. To more intuitively understand the differences in the ALP activity of bone repair materials, ALP activity was detected (Fig. 4B). Compared with that of the control group, the ALP activities of Bru-CPCs, Bru-CPCs/1.5% MMT, and Bru-CPCs/1.5% MMT@PDA are all increased.

The effect of the addition of MMT and MMT@PDA on cell osteogenic differentiation was further studied by detecting the messenger RNA expression levels of 5 related osteogenic genes (Col-I, IBSP, OCN, OPN, and Runx2). The expressions of Col-I, IBSP, OCN, OPN, and Runx2 of MC3T3-E1 after culturing with Bru-CPCs, Bru-CPCs/1.5% MMT, and Bru-CPCs/1.5% MMT@PDA are shown in Fig. 4C. After culturing for 7 d, the expression level of osteogenesis-related genes (Col-I, IBSP, and OCN) in the Bru-CPCs/1.5% MMT@PDA group had significant difference with those of the other groups. Based on the higher expression levels of the 3 genes, it could be seen that the addition of MMT@PDA into bone repair materials might costimulate the signaling pathways of MC3T3-E1 osteogenic differentiation and promote bone repair.

## Discussion

Although Bru-CPCs exhibit favorable characteristics for clinical applications, such as excellent biocompatibility and osteoconductivity, their mechanical properties remain suboptimal and require further enhancement to meet the demands of load-bearing bone repair [10,28]. MMT, a silicate nanoclay with a 2-dimensional layered structure, has been widely recognized for its potential to improve the mechanical properties of biomaterials, particularly in polymer-based scaffolds, due to its ability to enhance interfacial interactions between organic and inorganic phases [25,29]. Inspired by these findings, MMT-based Bru-CPCs (Bru-CPCs/1.5% MMT) was fabricated in this study. The compressive strength of Bru-CPCs was 8.46 MPa. The addition of MMT increased the compressive strength of Bru-CPCs/1.5% MMT by 57% (13.31 MPa) when compared to the one of Bru-CPCs. The result was not yet satisfactory. The limited enhancement in mechanical properties in this study may be attributed to the inherent challenges associated with interfacial interactions between MMT and inorganic materials (inorganic–inorganic phase), which differ from the strong interfacial bonding observed in MMT–polymer composites (organic–inorganic phase) [30–33]. This discrepancy highlights the need for further investigation into optimizing the interfacial compatibility and bonding mechanisms between MMT and inorganic matrices in CPCs. Therefore, surface modification techniques, such as functionalization of MMT or the incorporation of interfacial coupling agents, to strengthen the interaction between MMT and the inorganic phase, thereby further improving the mechanical performance of MMT-reinforced Bru-CPCs.

Extensive studies have demonstrated that the polymerization of dopamine introduced to coat inorganic materials can provide rich and dense interfacial interaction (covalent and hydrogen bonds, electrostatic interaction, and π conjugation) and improve the mechanical properties [34–37]. Thus, MMT was coated with a PDA layer to get MMT@PDA. The addition of MMT@PDA further increased the compressive strength of Bru-CPCs/1.5% MMT@PDA by 96% (16.58 MPa) when compared to the one of Bru-CPCs. This result, owing to the surface modification of MMT by PDA, enhanced the interfacial interactions and increased the compressive strength of CPCs/1.5% MMT@PDA. Moreover, with the introduction of MMT@PDA, the crystals of Bru-CPCs/1.5% MMT@PDA are more closely and regularly stacked, and the grain size becomes smaller, which is beneficial to improving the mechanical properties.

PDA is a pigment in natural melanin, which contains catechol, quinone, imine, amine functional groups, and π-conjugated structures [38]. The π-conjugated, wet-adhesive, and high-energy interface of PDA can combine with various guest molecules, including drugs, polymers, proteins, and nucleic acids, which may further achieve desirable bioapplications [39–41]. MC3T3-E1 cells are a well-established preosteoblastic cell line derived from mouse calvaria, which have been extensively used as a model system to study osteoblast differentiation, mineralization, and bone formation mechanisms. They have the ability to mimic key aspects of osteoblast behavior, including proliferation, extracellular matrix synthesis, and response to osteogenic stimuli. Hence, MC3T3-E1 cells provide a reliable model to assess whether our materials (MMT or MMT@PDA) can enhance osteogenic differentiation, which are critical for bone repair in osteoporotic conditions. Factors such as ALP, Col-I, IBSP, OCN, OPN, and Runx2 are critical in bone formation. PDA can up-regulate the expression of these factors, thereby promoting osteogenic differentiation [42–44]. Therefore, in our experiments, Bru-CPCs/1.5% MMT@PDA was constructed as a novel scaffold material and in vitro studies have shown that Bru-CPCs/1.5% MMT@PDA had no side effects on cell proliferation and up-regulated the expression of osteogenic relative gene markers to promote bone formation.

There are certain limitations to the present study. Firstly, MC3T3-E1 cells, a preosteoblastic cell line, were chosen to evaluate the biological effects in this study. Although these cells are widely used as a model for osteogenic activity, bone repair is a multicellular process involving dynamic interactions among osteoblasts, osteoclasts, endothelial cells, immune cells, and other cell types [45–47]. Thus, the effects of CPC samples on other cell types relevant to bone regeneration warrant further investigation. Secondly, before advancing to clinical applications, comprehensive in vivo studies using appropriate animal models are essential to evaluate the efficacy, biocompatibility, and long-term safety of CPC samples as a bone defect repair scaffold. These studies will provide critical insights into the material’s performance in a biologically relevant environment and its potential for clinical translation [48,49]. Lastly, the preparation of CPCs in this study was conducted on a small laboratory scale. For clinical application, large-scale production of the cement must be optimized, particularly with regard to the manufacturing process. Special attention should be paid to the exothermic nature of the setting reaction, as uncontrolled heat generation during curing could adversely affect both the material properties and surrounding tissues [50]. Addressing these limitations will be the focus of our future research, with the ultimate goal of developing a clinically viable bone repair material that meets the mechanical, biological, and practical requirements for bone defect regeneration.

In summary, this work aimed to modify the interfacial interactions between MMT and Bru-CPCs, thereby improving the mechanical properties and osteogenic bioactivity of MMT-based Bru-CPCs. MMT was coated with a PDA layer to get MMT@PDA, and then bone repair materials (Bru-CPCs/1.5% MMT and Bru-CPCs/1.5% MMT@PDA) with MMT and MMT@PDA as additives were fabricated. The results demonstrated that the addition of MMT and MMT@PDA affected the physicochemical properties and in vitro osteogenic differentiation ability of bone repair materials. Notably, the Bru-CPCs/1.5% MMT@PDA group exhibited the highest compressive strength, along with enhanced cell proliferation and osteogenic differentiation properties, compared to those of the other groups. This work provides an efficient and universal strategy to design and construct high-performance personalized materials for bone tissue repair.

## Data Availability

All data needed to evaluate the conclusions in the paper are present in the paper.
